# MUC1-C induces DNA methyltransferase 1 and represses tumor suppressor genes in acute myeloid leukemia

**DOI:** 10.18632/oncotarget.9777

**Published:** 2016-06-01

**Authors:** Ashujit Tagde, Hasan Rajabi, Dina Stroopinsky, Reddy Gali, Maroof Alam, Audrey Bouillez, Surender Kharbanda, Richard Stone, David Avigan, Donald Kufe

**Affiliations:** ^1^ Department of Medical Oncology, Dana-Farber Cancer Institute, Harvard Medical School, Boston, MA, USA; ^2^ Beth Israel Deaconess Medical Center, Harvard Medical School, Boston, MA, USA; ^3^ Department of Biomedical Informatics, Harvard Medical School, Boston, MA, USA

**Keywords:** MUC1-C, DNMT1, DNA methylation, decitabine, CDH1

## Abstract

Aberrant DNA methylation is a hallmark of acute myeloid leukemia (AML); however, the regulation of DNA methyltransferase 1 (DNMT1), which is responsible for maintenance of DNA methylation patterns, has largely remained elusive. MUC1-C is a transmembrane oncoprotein that is aberrantly expressed in AML stem-like cells. The present studies demonstrate that targeting MUC1-C with silencing or a pharmacologic inhibitor GO-203 suppresses DNMT1 expression. In addition, MUC1 expression positively correlates with that of DNMT1 in primary AML cells, particularly the CD34+/CD38− population. The mechanistic basis for this relationship is supported by the demonstration that MUC1-C activates the NF-κB p65 pathway, promotes occupancy of the MUC1-C/NF-κB complex on the *DNMT1* promoter and drives *DNMT1* transcription. We also show that targeting MUC1-C substantially reduces gene promoter-specific DNA methylation, and derepresses expression of tumor suppressor genes, including *CDH1*, *PTEN* and *BRCA1*. In support of these results, we demonstrate that combining GO-203 with the DNMT1 inhibitor decitabine is highly effective in reducing DNMT1 levels and decreasing AML cell survival. These findings indicate that (i) MUC1-C is an attractive target for the epigentic reprogramming of AML cells, and (ii) targeting MUC1-C in combination with decitabine is a potentially effective clinical approach for the treatment of AML.

## INTRODUCTION

Acute myeloid leukemia (AML) is a heterogeneous clonal hematopoietic stem cell disorder consisting of diverse genomic and epigenomic landscapes [1]. Mutations in one of nine categories of genes have been linked to the pathogenesis of AML, including those involving *NPM1* and *CEBPA*, among others [1, 2]. Studies of genome-wide methylation in AML have further demonstrated that such driving genetic mutations are associated with common sets of aberrantly methylated genes [1, 3, 4]. Whole genome and exome sequencing studies of AML have also identified mutations in genes encoding proteins involved in the epigenetic regulation of transcription [1]. For instance, recurrent mutations have been found in genes encoding DNA methyltransferase 3a (DNMT3a), ten-eleven translocation 2 (TET2) and isocitrate dehydrogenase 1/2 (IDH1/IDH2) that contribute to AML pathogenesis by aberrantly regulating DNA methylation of tumor suppressor genes (TSGs) [1, 5–9]. In this context, repression of the tumor suppressor *CDH1* gene by promoter hypermethylation [10, 11] is associated with an unfavorable prognosis in AML patients [12]. These findings have emphasized the potential of using epigenetically targeted therapies for the treatment of AML. Indeed, the DNMT inhibitors 5-azacitidine and 5-aza-2′deoxycytidine (decitabine) are effective agents in the treatment of patients with myelodysplastic syndromes and AML [13].

DNA methylation is mediated by DNA methyltransferses (DNMTs), including DNMT1, DNMT3a and DNMT3b. DNMT1 maintains DNA methylation patterns, whereas DNMT3a and DNMT3b establish de-novo methylation on nascent DNA strands [14, 15]. In this way, DNMT1 localizes at replication foci during S phase of the cell cycle and thereby plays a dominant role in maintaining global and gene specific CpG methylation [16]. Notably, the conditional knockout of *DNMT1* hinders leukemia development [17]. Haploinsufficiency of DNMT1 also delays leukemia progression and inhibits self-renewal of leukemia stem cells [17]. Additionally, DNMT1 haploinsufficiency is associated with decreases in DNA methylation and derepression of TSGs [17]. The anti-leukemic agent decitabine downregulates DNMT1, but has little effect on DNMT3a and DNMT3b expression [18]. These findings have collectively supported the importance of DNMT1 in maintaining DNA methylation patterns in AML cells.

Mucin 1 (MUC1) is a heterodimeric protein that is aberrantly expressed in AML blasts [19, 20] and AML stem cells [21]. The functional role for MUC1 in AML is not well defined; however, studies of the two MUC1 subunits have provided insights into a role for MUC1 in activation of intracellular signaling pathways [22]. In this respect, the extracellular N-terminal subunit (MUC1-N) contains glycosylated tandem repeats that are a characteristic of the mucin family [23]. MUC1-N forms a cell surface complex with the transmembrane C-terminal subunit (MUC1-C). MUC1-C functions as an oncoprotein in part by interacting with receptor tyrosine kinases (RTKs), such as FLT3, at the cell membrane and promoting activation of their downstream pathways [24]. In this way, the intrinsically disordered MUC1-C cytoplasmic domain is phosphorylated by RTKs and other kinases, and thereby interacts with effectors that have been linked to transformation [23, 25, 26]. MUC1-C is also imported into the nucleus, where it interacts with transcription factors, such as NF-κB p65, and contributes to activation of their target genes [27, 28]. The MUC1-C cytoplasmic domain also contains a CQC motif that is necessary for MUC1-C homodimerization and nuclear localization [25, 27, 29]. Accordingly, the cell-penetrating peptide GO-203 was developed to target the CQC motif and block MUC1-C homodimerization and function [22]. Treatment of AML cells with GO-203 is associated with arrest of growth and induction of terminal differentiation [22]. In addition, GO-203 is effective in treating human AML established in NSG mice, but has no apparent effect on engraftment of normal hematopoietic cells [21]. These findings have provided support for MUC1-C as a target in AML treatment.

The present study demonstrates that MUC1-C regulates DNMT1 expression and thereby DNA methylation of TSGs in AML cells. We show that MUC1-C induces *DNMT1* gene transcription by an NF-κB p65-dependent mechanism and that MUC1 expression correlates significantly with that for DNMT1 in primary CD34+/CD38− AML cells. The results also demonstrate that targeting MUC1-C with GO-203 in combination with decitabine is more effective in suppressing both DNMT1 and AML cell survival than either agent alone.

## RESULTS

### Targeting MUC1-C downregulates DNMT1 expression in AML cells

**Figure 1:**
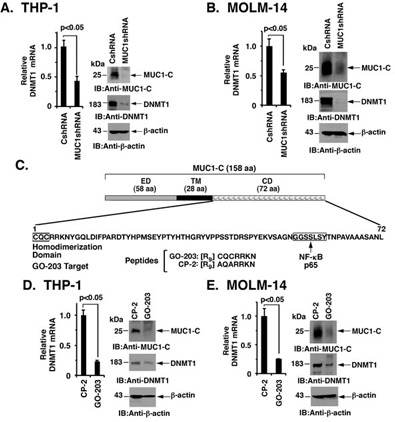
MUC1-C drives DNMT1 expression **A.**-**B.** THP-1 **A.** and MOLM-14 **B.** AML cells were transduced with lentiviral vectors to stably express a control shRNA (CshRNA) or a MUC1 shRNA. DNMT1 mRNA levels were determined using qRT-PCR. The results (mean±SD of three determinations) are expressed as a relative DNMT1 mRNA levels as compared to that obtained with CshRNA cells (assigned a value of 1) (left). Forward and reverse primers are listed in Supplemental Table S1. Lysates from the indicated cells were immunoblotted with antibodies against MUC1-C, DNMT1 and β-actin (right). **C.** Schema of the MUC1-C subunit with a 58 amino acid (aa) extracellular domain (ED) and the 28 aa transmembrane domain (TM). The sequence of the 72 aa cytoplasmic domain (CD) is highlighted at the CQCRRKN motif, which is targeted by the cell penetrating GO-203 peptide and not the control CP-2 peptide. Also highlighted is the NF-κB p65 binding site. **D.**-**E.** THP-1 and MOLM-14 cells were treated with 5 μM of GO-203 or CP-2 for 72 h. DNMT1 mRNA levels were determined using qRT-PCR. The results (mean±SD of three determinations) are expressed as a relative DNMT1 mRNA levels as compared to that obtained with CP-2-treated cells (assigned a value of 1) (left). Lysates were immunoblotted with the indicated antibodies (right).

To investigate the potential role of MUC1-C in the regulation of DNMT1 expression, we first stably silenced MUC1-C in THP-1 cells. Downregulation of MUC1-C significantly reduced DNMT1 mRNA levels in THP-1/MUC1shRNA cells as compared to control THP-1/CshRNA cells (Figure 1A, left). In concert with these results, DNMT1 protein was also suppressed in response to MUC1-C silencing (Figure 1A, right). Similarly, stable silencing of MUC1-C in MOLM-14 cells was associated with significant decreases in DNMT1 mRNA and protein levels (Figure 1B, left and right). The MUC1-C cytoplasmic domain includes a CQC motif, which is essential for the formation of MUC1-C homodimers and thereby the MUC1-C oncogenic function (Figure 1C). Accordingly, we treated AML cells with GO-203, which is a cell penetrating peptide that contains a poly-Arg cell transduction domain linked to CQCRRKN (Figure 1C). GO-203 binds to the corresponding endogenous sequence in the MUC1-C cytoplasmic domain and blocks MUC1-C homodimerization [25, 29]. As a control, we used the peptide CP-2, which is inactive in targeting the MUC1-C CQC motif. Treatment of THP-1 cells with GO-203, but not CP-2, downregulated MUC1-C expression and also reduced DNMT1 mRNA and protein levels (Figure 1D, left and right). Similar results were obtained in MOLM-14 cells, such that expression of MUC1-C and DNMT1 was downregulated by GO-203 and not CP-2 treatment (Figure 1E, left and right).

### MUC1 correlates with DNMT1 expression in primary AML cells

**Figure 2:**
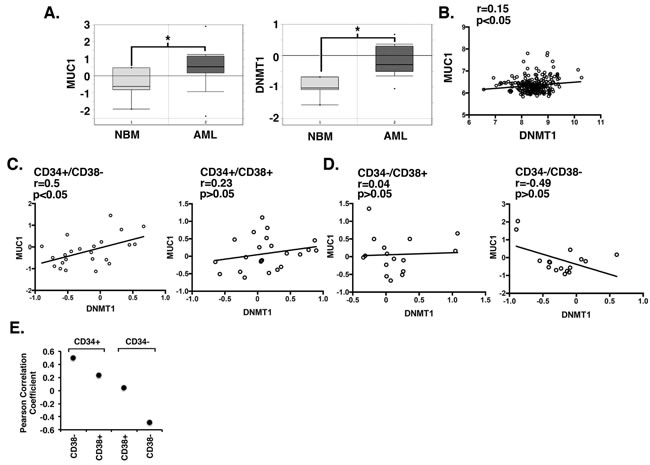
MUC1 correlates with DNMT1 expression in primary AML cells **A.** Microarray data obtained from the Oncomine database (GSE7186) is expressed as box plots for MUC1 (left) and DNMT1 (right) in normal bone marrow (NBM, *n* = 6) and AML (*n* = 22) cells. The data was log2 transformed and median centered (dark lines). The asterisk denotes a p value of < 0.05. **B.**-**D.**. Microarray gene expression data from GEO database GSE17855 **B.** and GSE30375 **C.**-**D.** was RMA normalized and the correlation between MUC1 and DNMT1 expression in AML patients was assessed by Pearson's correlation coefficient. **E.** Correlation between MUC1 and DNMT1 expression in the indicated CD34 and CD38 AML cell phenotypes.

Based on the above findings, we analyzed an Oncomine dataset derived from AML cells to assess the association between MUC1 and DNMT1 expression. In concert with our previous studies [21], MUC1 expression was significantly elevated in AML cells (*n* = 22) as compared to that in normal bone marrow cells (*n* = 6) (Figure 2A, left). We also found that, like MUC1, DNMT1 expression is increased in AML cells (Figure 2A, right). To determine whether expression of MUC1 and DNMT1 correlate, we performed bioinformatics analysis on GEO microarray datasets. Analysis of GSE17855 (*n* = 237) demonstrated that MUC1 and DNMT1 expression correlate positively (Figure 2B). To further investigate the nature of this relationship, we analyzed the microarray dataset (GSE30375) in which AML cells were sorted according to expression of CD34 and CD38. Interestingly, there was a strong positive correlation (Pearson r = 0.50; *p* < 0.05) between MUC1 and DNMT1 in CD34+/CD38− AML cells (Figure 2C, left). However, the correlation was less pronounced in CD34+/CD38+ cells (Pearson r = 0.23) (Figure 2C, right). Moreover and in contrast, no correlation was found in CD34-/CD38+ cells (Pearson r = 0.04) and a negative correlation was obtained in CD34−/CD38− cells (Pearson r = −0.49) (Figure 2D, left and right). Together, these results indicate that MUC1 positively correlates with DNMT1 in CD34+/CD38−, but not in CD34-/CD38−, AML cells (Figure 2E).

### MUC1-C upregulates DNMT1 expression by an NF-κB p65-dependent mechanism

**Figure 3:**
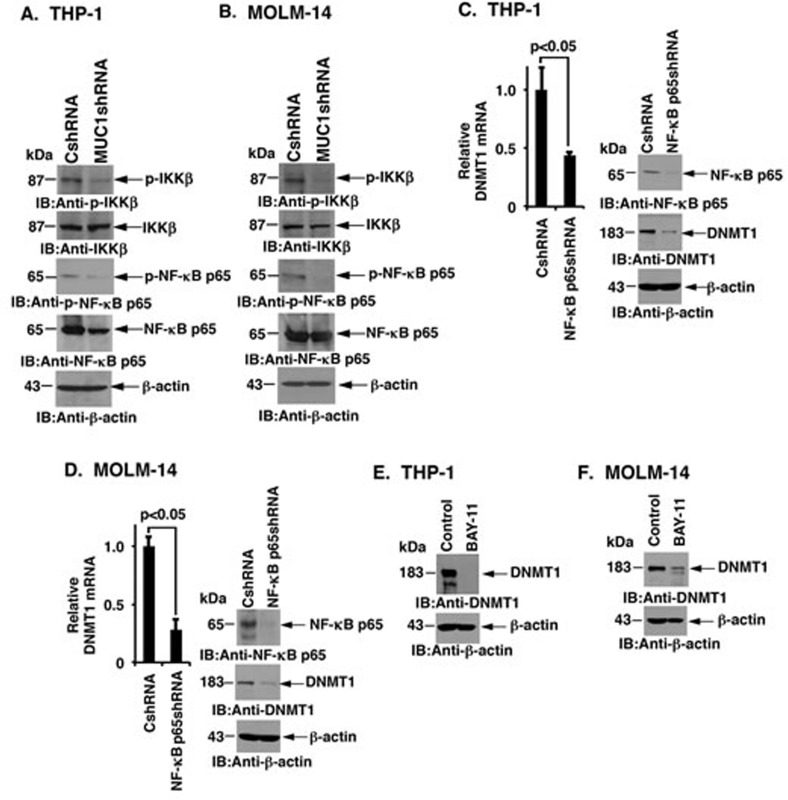
MUC1-C induces DNMT1 expression by an NF-κB p65 dependent mechanism **A.**-**B.** Lysates from THP-1 **A.** and MOLM-14 **B.** cells were subjected to immunoblot analysis with the indicated antibodies. **C.**-**D.** THP-1 **C.** and MOLM-14 **D.** cells were transduced with lentiviral vectors to stably express a control shRNA (CshRNA) or a NF-κB p65 shRNA. DNMT1 mRNA levels were determined using qRT-PCR. The results (mean±SD of three determinations) are expressed as a relative DNMT1 mRNA levels as compared to that obtained with CshRNA cells (assigned a value of 1) (left). Lysates from the indicated cells were immunoblotted with antibodies against NF-κB p65, DNMT1 and β-actin (right). **E.**-**F.** THP-1 **E.** and MOLM-14 **F.** cells were treated with control vehicle or BAY-11-7085 (left). Lysates from the control and BAY-11-7085 treated cells were immunoblotted with the indicated antibodies (right).

Studies in human carcinoma cells have shown that the MUC1-C cytoplasmic domain activates the NF-κB p65 pathway by interacting directly with IKKs and NF-κB p65 (Figure 1C) [28, 30]. Silencing MUC1-C in THP-1 cells reduced phospho-IKKβ and phospho-NF-κB p65, but had less pronounced effects on IKKβ or NF-κB levels, indicating that MUC1-C is also of importance for activation of the IKK→NF-κB pathway in AML cells (Figure 3A). Silencing MUC1-C in MOLM-14 cells was also associated with downregulation of phospho-IKK and phospho-NF-κB p65 (Figure 3B). Consistent in part with a study showing that nucleolin regulates DNMT1 expression by an NF-κB dependent pathway [31], we found that silencing NF-κB p65 in THP-1 cells is associated with significant decreases in DNMT1 mRNA and protein (Figure 3C, left and right). Similar results were obtained in MOLM-14 cells, such that DNMT1 levels were significantly reduced after silencing NF-κB p65 (Figure 3D, left and right). Furthermore, treatment of THP-1 and MOLM-14 cells with the small molecule NF-κB inhibitor BAY-11-7085 suppressed DNMT1 levels (Figure 3E and 3F). These results collectively indicated that DNMT1 expression is regulated in AML cells, at least in part, by the MUC1-C→NF-κB pathway.

### MUC1-C activates the *DNMT1* promoter in a complex with NF-κB p65

**Figure 4:**
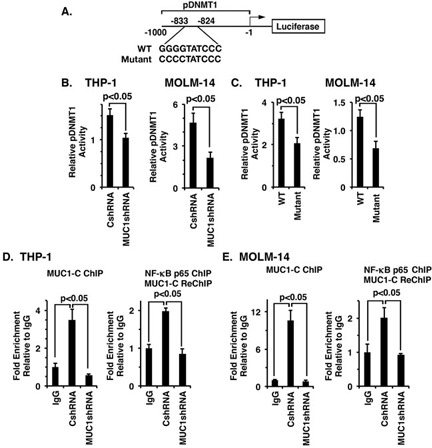
Targeting MUC1-C suppresses *DNMT1* transcription **A.** Schema of the pDNMT1-Luc reporter with the positioning of the NF-κB binding site (−833 to −824) upstream of the transcription start site. **B.** The indicated THP-1 (left) and MOLM-14 (right) cells were transfected with (i) the empty pGL3-Basic Luc vector or pGL3-pDNMT1-Luc, and the SV-40-*Renilla*-Luc plasmid as an internal control. Dual luciferase activity was measured at 24 h after transfection. The results (mean±SD of 3 determinations) are expressed as the relative luciferase activity compared to that obtained with pGL3-Basic Luc. **C.** THP-1 (left) and MOLM-14 (right) cells were transfected with the empty pGL3-Basic Luc vector, wild-type (WT) pGL3-pDNMT1-Luc or mutant pGL3-pDNMT1-Luc and the SV-40-*Renilla*-Luc plasmid. Dual luciferase activity was measured at 24 h after transfection. The results (mean±SD of 3 determinations) are expressed as the relative luciferase activity compared to that obtained with pGL3-Basic Luc. **D.**-**E.** Soluble chromatin from the indicated THP-1 **D.** and MOLM-14 **E.** cells was precipitated with anti-MUC1-C or a control IgG (left). In the re-ChIP experiments, NF-κB p65 precipitates were released and re-immunoprecipitated with anti-MUC1-C and a control IgG (right). The final DNA samples were amplified by qPCR with primers for the *DNMT1* promoter or as a control *GAPDH (*Supplemental Table. S2). The results (mean±SD of three determinations) are expressed as the relative fold enrichment compared with that obtained with the IgG control.

To investigate if MUC1-C activates the *DNMT1* promoter in AML cells, we transfected THP-1/CshRNA and THP-1/MUC1shRNA cells with a pDNMT1-Luc reporter that contains a consensus NF-κB binding site (GGGGTATCCC) at positions −833 to −824 upstream of the transcription start site (Figure 4A). Silencing MUC1-C in THP-1 cells was associated with a decrease in pDNMT1-Luc activity as compared to that in THP-1/CshRNA cells (Figure 4B, left). Similar results were obtained in MOLM-14/MUC1shRNA cells with pDNMT1-Luc activity significantly lower as compared to that in MOLM-14/CshRNA cells (Figure 4B, right). In addition, mutation of the putative NF-κB p65 binding site in the pDNMT1-Luc vector (Figure 4A) decreased activation in THP-1 and MOLM-14 cells (Figure 4C, left and right). ChIP studies on soluble chromatin from THP-1 cells further demonstrated that MUC1-C occupancy is detectable on the *DNMT1* promoter (Figure 4D, left). Additionally, re-ChIP analysis demonstrated the presence of MUC1-C and NF-κB p65 complexes (Figure 4D, right). Consistent with these results, silencing MUC1-C in THP-1 cells significantly reduced occupancy of MUC1-C and MUC1-C/NF-κB p65 complexes on the *DNMT1* promoter (Figure 4D, left and right). Studies of MOLM-14 cells also demonstrated that MUC1-C forms complexes with NF-κB p65 on the *DNMT1* promoter and that silencing MUC1-C reduces their occupancy (Figure 4E, left and right). Together, these findings support a model in which MUC1-C occupies the *DNMT1* promoter in a complex with NF-κB p65 and drives *DNMT1* transcription.

### MUC1-C induces DNA methylation of TSGs

**Figure 5:**
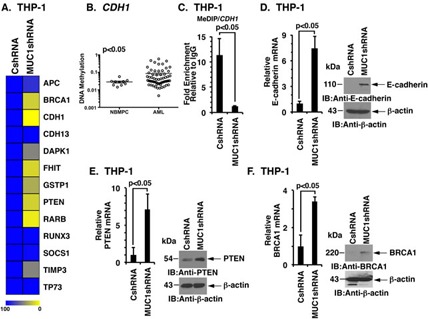
MUC1-C regulates DNA methylation in AML cells **A.** Heatmap showing relative methylation levels of the indicated TSG promoters in THP-1/CshRNA and THP-1/MUC1shRNA cells. **B.** The GSE58477 dataset from GEO was analyzed to assess the methylation of CpG islands in the *CDH1* gene promoter in AML cells (*n* = 62) as compared to normal bone marrow progenitor cells (NBMPCs) cells (*n* = 10). The open circles indicate the β-value in the logarithmic scale. The horizontal bars indicate mean±SEM and *p* < 0.05 indicates statistical difference in the mean. **C.** The *CDH1* promoter in the indicated THP-1 cells was analyzed for CpG methylation using the MeDIP assay. The results (mean±SD of three determinations) are expressed as the relative fold enrichment compared with that obtained with the IgG control. **D.** The indicated THP-1 cells were analyzed for E-cadherin mRNA levels by qRT-PCR (left). The results are expressed as a relative E-cadherin mRNA levels (mean±SD of three determinations) as compared to that obtained with CshRNA cells (assigned a value of 1). Lysates from THP-1/CshRNA and THP-1/MUC1shRNA cells were immunoblotted for indicated antibodies (right). (E-F) The indicated THP-1 cells were analyzed for PTEN **E.** and BRCA1 **F.** mRNA levels by qRT-PCR (left). The results are expressed as a relative mRNA levels (mean±SD of three determinations) as compared to that obtained with CshRNA cells (assigned a value of 1). Lysates from THP-1/CshRNA and THP-1/MUC1shRNA cells were immunoblotted for indicated antibodies (right).

The observation that MUC1-C induces DNMT1 expression in AML cells invoked the possibility that MUC1-C drives DNA methylation of TSGs. In this context, silencing MUC1-C in THP-1 cells was associated with decreased DNA methylation of certain TSG promoters (Figure 5A). Notably, DNA methylation of *CDH1* is increased in AML cells as compared to that in normal progenitor cells (Figure 5B). We therefore confirmed the effects of silencing MUC1-C on methylation of CpG islands in the *CDH1* promoter. The results demonstrate that the fraction of methylated CpG islands is significantly lower in THP-1/MUC1shRNA as compared to that in THP-1/CshRNA cells (Figure 5C). Moreover and in concert with the decrease in *CDH1* promoter methylation, we found that targeting MUC1-C is associated with derepression of *CDH1* and thereby upregulation of E-cadherin mRNA and protein (Figure 5D, left and right). Silencing MUC1-C also resulted in marked derepression of *PTEN* and *BRCA1* expression (Figure 5E and 5F, left and right; Supplemental Figure S1A and S1B, left and right).

### GO-203 is synergistic with decitabine in downregulating DNMT1 and in the treatment of AML cell lines

**Figure 6:**
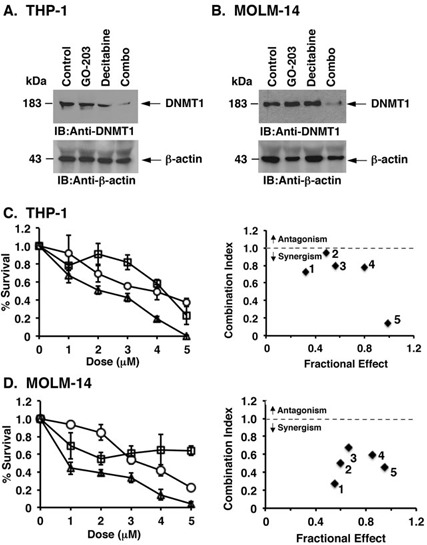
GO-203 is synergistic with decitabine in downregulating DNMT1 expression and in the treatment of AML cells **A.**-**B.** THP-1 **A.** and MOLM-14 **B.** cells were left untreated (Control) and treated with 2 μM CP-2, 2 μM GO-203, 2 μM decitabine or the combination for 48 h. Lysates were immunoblotted with the indicated antibodies. **C.**-**D.** THP-1 **C.** and MOLM-14 **D.** cells were treated with the indicated concentrations of GO-203 (open circles) or decitabine (open squares) or the combination (open triangles) for 96 h. Cell viability was assessed by the Cell Titer-Glo Luminescent viability assay. The results are expressed as percentage survival (mean±SD of three determinations) (left). Combination Index (CI) numbers were calculated for the fixed ratio of GO-203 and decitabine (1:1) using CompuSyn software (right). The CI values < 1 indicate synergism and > 1 indicate antagonism.

The demonstration that targeting MUC1-C downregulates DNMT1 expression suggested that GO-203 could be effective in combination with decitabine, a hypomethylating agent that also downregulates DNMT1 [18]. Indeed, treatment of THP-1 cells with low doses of GO-203 and decitabine in combination was more effective in downregulating DNMT1 expression than that found with either agent alone (Figure 6A). Similar results were obtained with MOLM-14 cells (Figure 6B), indicating that the effects of combining low dose GO-203 and decitabline are potentially synergistic. In concert with this notion, combining GO-203 with decitabine synergistically reduced survival of THP-1 cells, as supported by combination index (CI) values < 0.7 (Figure 6C, left and right). Treatment of MOLM-14 cells with GO-203 and decitabine also induced synergistic cell death (CI < 0.7)(Figure 6D, left and right).

### Effects of targeting MUC1-C on primary AML cells

**Figure 7:**
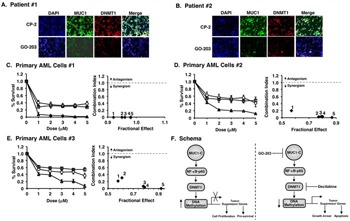
Targeting MUC1-C with GO-203 downregulates DNMT1 expression and synergistically enhances decitabine induced cell death in primary AML cells **A.**-**B.** Primary AML cells from patients #1 **A.** and #2 **B.** were treated with 5μM of CP-2 or GO-203 for 72 h and then probed for MUC1-C (green) and DNMT1 (red) expression. DAPI counterstained was used to visualize nuclei. Immunofluorescence images were captured using Nikon Ti inverted microscope at a magnification of 40X. **C.**-**E.** Primary AML sample #1 **C.**, primary AML sample #2 **D.** and primary AML sample #3 **E.** cells were treated with the indicated concentrations of GO-203 (open circles), decitabine (open squares) or the combination (open triangles) for 96 h. Cell viability was measured by the Cell Titer-Glo Luminescent viability assay. The results are expressed as percentage survival (mean±SD of three determinations)(left). Combination Index (CI) numbers were calculated for fixed ratio of GO-203 and decitabine (1:1) using CompuSyn software. The CI values < 1 indicate synergism and > 1 indicate antagonism (right). **F.** Schematic representation of MUC1-C induced regulation of DNMT1 and DNA methylation. MUC1-C upregulates DNMT1 expression by an NF-κB p65-dependent mechanism and thereby increases DNA methylation of TSGs (left). In this way, MUC1-C represses TSGs and promotes proliferation and survival. Targeting AML cells with GO-203 and decitabine synergistically downregulates DNMT1 expression and thereby derepresses TSG expression leading to growth arrest and apoptosis (right).

To extend our results obtained with AML cell lines, we treated primary AML cells with GO-203 and CP-2. In samples from patients #1 and #2, we found that treatment with GO-203 is associated with significant downregulation of both MUC1-C and DNMT1 (Figure 7A and 7B). Additionally, combining GO-203 and decitabine induced highly synergistic cell death in primary AML samples #1 (Figure 7C, left and right), #2 (Figure 7D, left and right) and #3 (Figure 7E, left and right).

## DISCUSSION

Genome-wide studies in AML have shown that common sets of genes are subject to aberrant DNA methylation patterns [1, 3, 4]. In addition, distinct DNA methylation profiles have been identified in AML with specific genetic alterations, such as PML-RARα and AML1-ETO, among others [3]. Mutations in genes involved in the regulation of DNA methylation and hydroxylation, such as DNMT3a, TET2 and IDH1/2, have also been identified in AML cells [8, 9, 32, 33]. The present findings extend this list to the oncogenic MUC1-C protein, which is overexpressed and, unlike DNMT3a, TET2 and IDH1/2, is not known to be mutated in AML and other malignancies. Surprisingly for an epithelial cell-associated apical membrane protein [23, 34], MUC1-C is aberrantly expressed in AML and is associated with the self-renewing AML stem-like cell population that has the capacity to engraft leukemia in NSG mice [21]. Additionally, targeting MUC1-C in AML cells is associated with loss of self-renewal capabilities and the induction of terminal differentiation [22]. Given these findings, we investigated the notion that MUC1-C may play a role in the epigenetic regulation of gene expression in AML cells. Indeed, we found that targeting MUC1-C is linked to downregulation of DNMT1, which is essential for maintaining DNA methylation and thereby survival of human stem cells [35]. Our results thus provide support for the novel finding that the MUC1-C drives DNMT1 expression in AML cells.

DNMT1 expression is regulated by diverse mechanisms, including activation of *DNMT1* transcription by c-JUN, pRb/E2F1, Sp1, Sp3 and NF-κB [31, 36–38]. DNMT1 mRNA levels are also suppressed by certain miRNAs, such as miR-148b and miR-152 [39]. In this way, the response to targeting MUC1-C with downregulation of DNMT1 mRNA and protein could be mediated by multiple pathways. The MUC1-C cytoplasmic domain is an intrinsically disordered protein, a characteristic found in other oncoproteins that integrate the signaling of diverse effectors that are associated with transformation [25]. In concert with this structure, the MUC1-C cytoplasmic domain interacts directly with PI3K, STAT1/3, β-catenin and GRB2, among others [26]. The MUC1-C cytoplasmic domain also intersects with the TAK1→IKK→NF-κB p65 pathway, linking an inflammatory circuit with changes in gene expression [28, 30, 40]. The present results demonstrate that the MUC1-C→NF-κB p65 pathway drives *DNMT1* transcription in AML cells. Consistent with certain other NF-κB p65 target genes, including *MUC1* itself [28], we found that MUC1-C occupies the *DNMT1* promoter in a complex with NF-κB p65 and promotes NF-κB-mediated *DNMT1* transcription. These results thus support a model in which the MUC1-C→NF-κB p65 autoinductive inflammatory circuit contributes to the activation of DNMT1 expression in AML cells (Figure 7F). This model is further supported by the analysis of AML datasets that demonstrated (i) increased expression of MUC1 and DNMT1, and (ii) significant correlations between MUC1-C and DNMT1 expression, particularly in AML stem cells and not in more differentiated progenitors. These results and those obtained in AML cell lines provided convincing evidence that MUC1-C drives *DNMT1* transcription.

Silencing DNMT1 in embryonic stem cells results in rapid loss of DNA methylation [35]. To extend this observation, we studied TSG promoters in AML cells and found that targeting MUC1-C induces significant decreases in CpG methylation. Moreover and in concert with this response, targeting MUC1-C resulted in derepression of the *CDH1* gene and thereby upregulation of E-cadherin expression. We also found that targeting MUC1-C depresses expression of the *PTEN* and *BRCA1* genes. These results lend credence to the possibility that targeting MUC1-C in AML cells is associated with the induction of other TSGs, which will be a focus of subsequent studies. Interestingly, DNMT1 has also been linked to the epigenetic regulation of genes, such as *BRCA1*, that control DNA damage and repair, and thereby genomic stability [34]. Therefore, MUC1-C could be involved in the accumulation of mutagenic events in AML cells. Of note, our studies here on DNMT1 do not exclude the possibility that MUC1-C activates other genes that encode DNMTs, such as DNMT3a and/or DNMT3b. In this context, recent studies in human carcinoma cells have shown that MUC1-C drives transcription of *DNMT1* and *DNMT3b*, but not *DNMT3a* [51].

Our findings further support the potential of targeting MUC1-C in combination with DNMT inhibitors. Decitabine is approved by the European Medicines Agency, but not by the US FDA, for the treatment of adult patients with AML [41]. In this respect, decitabine is active in inducing complete remissions in patients with AML, but has a modest effect on overall survival, highlighting the need to improve the outcome of decitabine treatment [42, 43]. For this reason, we assessed the effects of combining GO-203 and decitabine and found that the combination is more effective in decreasing DNMT1 levels than that obtained with either agent alone. In addition, the GO-203/decitabine combination was found to confer synergistic killing of AML cell lines and primary AML blasts, indicating that targeting DNMT1 with GO-203 and decitabine is potentially a highly effective approach for the treatment of AML. Based on these results, we have initiated a Phase I/II trial of GO-203 in combination with decitabine for patients with relapsed/refractory AML.

## MATERIALS AND METHODS

### Cell culture

Human THP-1 (ATCC) and MOLM-14 (ATCC) cells were cultured in RPMI1640 medium supplemented with 10% heat-inactivated fetal bovine serum, 100 units/ml penicillin, 100 ug/ml streptomycin and 2 mM L-glutamine. Authenticity of the cells was confirmed by short tandem repeat (STR) analysis [44]. Cells were infected with lentiviral vectors expressing a MUC1 shRNA, NF-κB p65 shRNA or scrambled control shRNA vector (Sigma). Cells were selected and maintained in puromycin [40]. Cells were treated with the NF-κB pathway inhibitor BAY-11-7085 (Santa Cruz Biotechnology) or DMSO as a vehicle control.

### Immunoblot analysis

Cells were lysed using NP-40 lysis buffer containing protease cocktail inhibitor (Thermo Scientific), DTT and PMSF. Soluble proteins were analyzed by immunoblotting with anti-MUC1-C antibody (Thermo Scientific), anti-DNMT1 (Abcam), anti-NF-κB p65 (Santa Cruz Biotechnology), anti-phospho-NF-κB p65, anti-IKKβ, anti-phospho-IKKβ, anti-E-cadherin, anti-PTEN (Cell Signaling), anti-BRCA1 (Santa Cruz Biotechnology) and anti-β-actin (Sigma). Detection of immune complexes was achieved using horseradish peroxidase-conjugated secondary antibodies and enhanced chemuluminescence (GE Healthcare, Piscataway, NJ) [45, 46].

### Quantitative real-time, reverse-transcriptase PCR (qRT-PCR)

Quantitative qRT-PCR analysis was performed as described [47]. Briefly, total mRNA was isolated using the RNAeasy mini kit (Invitrogen). cDNA was synthesized using the High-Capacity cDNA Reverse Transcription Kit (Invitrogen). cDNA samples were then amplified using the SYBR Green qPCR assay kit (Applied Biosystem) and ABI Prism 7300 sequence detector (Applied Biosystem). qPCR primers for the detection of DNMT1, E-cadherin, PTEN, BRCA1 and β-actin mRNAs are listed in Supplementary Table S1. The results were analyzed using the delta delta Ct method as described [48]. Statistical significance was determined by the Student's *t*-test.

### *DNMT1* promoter-luciferase assays

Cells cultured in 96-well plates were transfected with the pGL3-Basic Luc vector, wild-type pDNMT1-Luc or mutant pDNMT1-Luc (GGGGTATCCC→CCCCTATCCC) and SV-40 *Renilla*-Luc in the presence of Lipofectamine 3000 Reagent (Invitrogen). At 24 h after transfection, the cells were lysed with passive lysis buffer and analyzed using the Dual Luciferase assay kit (Promega). Firefly luciferase values were normalized to that for *Renilla*-Luc. The normalized values for pDNMT1-Luc were then compared to that obtained with pGL3.

### Chromatin immunoprecipitation (ChIP) studies

Soluble chromatin was precipitated with anti-MUC1-C (Thermo Scientific) or a control non-immune IgG (Santa Cruz Biotechnology). For re-ChIP analysis, anti-NF-κB p65 (Santa Cruz Biotechnology) complexes from the primary ChIP were eluted and re-immunoprecipitated with anti-MUC1-C. For real-time ChIP qPCR, the SYBR green system was used with the ABI Prism 7300 sequence detector (Applied Biosystems). Data are reported as relative fold enrichment [49]. Primers used for ChIP qPCR of the *DNMT1* promoter and control regions are listed in the Supplementary Table S2.

### TSG promoter methylation analysis

The EpiTect Methyl II PCR System (Qiagen) was used to assess the methylation of TSG promoters according to the manufacturer's protocol.

### Methylated DNA immunoprecipitation (MeDIP)

For MeDIP analysis, genomic DNA was extracted using the DNAeasy kit (Qiagen), digested with MseI (300-1000 kbp fragments) and immunoprecipitated using an anti-5-methylcytidine (5-mC) antibody (Active Motif). The resulting enriched methylated DNA in the immunoprecipitated fraction was subjected to PCR analysis of *CDH1* promoter region using primers listed in Supplementary Table S2.

### Isolation of primary AML cells

Primary AML cells from patients were isolated as described [21]. Briefly, blood samples and bone marrow aspirates from AML patients were obtained according to an institutionally approved protocol. Mononuclear cells were isolated using Ficoll density centrifugation.

### Drug synergy studies

Cells were seeded in a 96-well plate and treated with GO-203 [47] and/or decitabine (Sigma). Cell viability was assessed using cell titer glow assay (Promega). Combination index values were determined by isobologram analysis using CompuSyn software. Combination index values < 1 were considered as synergistic and > 1 as antagonistic [44].

### Immunofluorescence imaging

Intracellular staining was performed as described [44, 47]. Briefly, primary AML cells were fixed with 2% paraformaldehyde and permeabilized with 100% methanol. Cells were then stained with anti-MUC1-C (Thermo Scientific) and anti-DNMT1 (Abcam), followed by incubation with secondary anti-hamster Alexa Fluor 488 and anti-rabbit Alexa Fluor 568 (Abcam) antibodies. Cells were counterstained with 4,6 diamidino-2-phenylindole (DAPI) and visualized using a Nikon Ti inverted microscope.

### Bioinformatic analyses

Datasets of AML patient samples were downloaded from gene expression omnibus (GEO) under the accession numbers GSE17855 and GSE30375. Data was RMA normalized (Bioconductor) and Log-2 transformed [47]. Multiple probes set IDs for MUC1 was averaged for each patient sample to obtain a representative expression value [40]. Correlations between MUC1 and DNMT1 expression were assessed by Pearson's coefficient analysis. Datasets were also downloaded from GEO under the accession number GSE58477 for assessment of methylation profiling by array [50]. The β-values for the *CDH1* (cg16739895) gene in AML (*n* = 62) and normal bone marrow progenitor cells (NBMPC, *n* = 10) were compared using the Student's *t*-test.

## SUPPLEMENTAL MATERIAL FIGURE AND TABLES


