# Supplementary material for: MUC1-C induces DNA methyltransferase 1 and represses tumor suppressor genes in acute myeloid leukemia
Source: Oncotarget. 2016 Jun 1;7(26):38974–87. doi: 10.18632/oncotarget.9777 (PMC5129907; doi:10.18632/oncotarget.9777)
Supplement: Supplementary file 1 [file oncotarget-07-38974-s001.pdf]

MUC1-C induces DNA methyltransferase 1 and represses tumor suppressor genes in acute myeloid leukemia

Supplemental Material

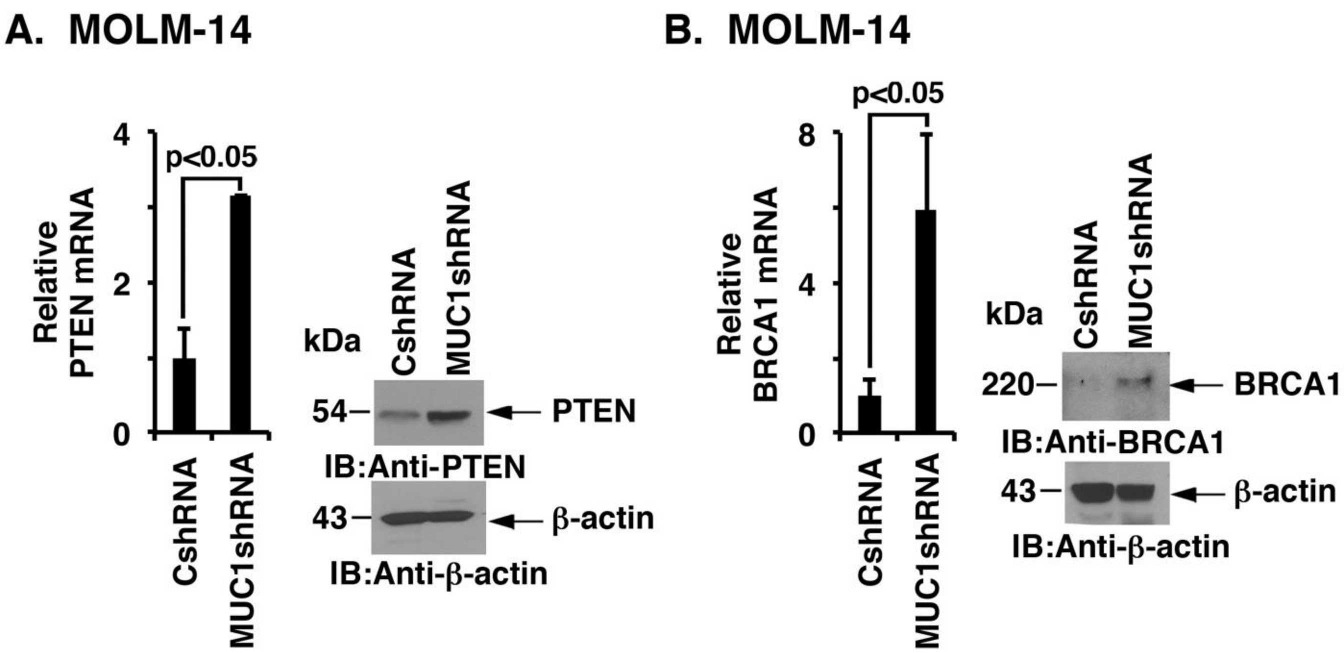

Supplemental Fig. S1. Silencing MUC1-C derepresses PTEN and BRCA1 expression in MOLM-14 cells. (A and B) The indicated MOLM-14 cells were analyzed for PTEN (A) and BRCA1 (B) mRNA levels by qRT-PCR (left). The results are expressed as a relative mRNA levels (mean±SD of three determinations) as compared to that obtained with CshRNA cells (assigned a value of 1). Lysates from MOLM-14/CshRNA and MOLM-14/MUC1shRNA cells were immunoblotted for indicated antibodies (right).

Supplemental Table S1. Primers used for qRT-PCR.

DNMT1	Fwd: 5′-CCCAAGTAACTGGGATTAGAGC-3′
DNMT1	Rev: 5′-GGTTTGCCTGGTGCTTTTC-3′
E-cadherin	Fwd: 5′-GAAGGTGACAGAGCCTCTGGAT-3′
E-cadherin	Rev: 5′- GATCGGTTACCGTGATCAAAATC-3′
BRCA1	Fwd: 5′-GCGTCCCCTCACAAATAAAT-3′
BRCA1	Rev: 5′- CTTGACCATTCTGCTCCGTT-3′
PTEN	Fwd: 5'-AAGGGACGAACTGGTGTAATG-3'
PTEN	Rev: 5'-GCCTCTGACTGGGAATAGTTA-3'
β-actin	Fwd: 5′-TTCTACAATGAGCTGCGTGTG-3′
β-actin	Rev: 5′-GGGGTGTTGAAGGTCTCAAA-3′

Supplemental Table S2. Primers used for ChIP and MeDIP qPCR.

DNMT1	Fwd: 5′-GCTAGGATTACAGGTGTGTGTC-3′
DNMT1	Rev: 5′-GATCGCTTGAGGTTAGGAGTTC-3′
CDH1	Fwd: 5′-CTTTCTGATCCCAGGTCTTAGTG-3′
CDH1	Rev: 5′-TAGGGTCTAGGTGGGTTATGG-3′
GAPDH	Fwd: 5′-TACTAGCGGTTTTACGGGCG-3′
GAPDH	Rev: 5′-TCGAACAGGAGGAGCAGAGAGCGA-3′
